# MicroRNA-mediated regulation of hair follicle regeneration: mechanisms, delivery strategies, and translational perspectives

**DOI:** 10.3389/fcell.2026.1880276

**Published:** 2026-06-26

**Authors:** Yuyu Deng, Kechun Hong, Mengting Xiong, Changxia Li, Xiaohua Tao

**Affiliations:** 1 Dermatology Hospital of Jiangxi Province, Nanchang, China; 2 Jiangxi Provincial Clinical Research Center for Skin Diseases, Nanchang, China; 3 Candidate Branch of National Clinical Research Center for Skin Diseases, Nanchang, China; 4 Dermatology Institute of Jiangxi Province, Nanchang, China; 5 The Affiliated Dermatology Hospital of Nanchang University, Nanchang, China

**Keywords:** alopecia, delivery systems, dermal papilla, hair follicle stem cells, microRNA, miRNA mimic

## Abstract

Hair regeneration depends on a complex interplay between the activation of hair follicle stem cells (HFSCs), maintenance of inductive capacity in the dermal papilla (DP), and the re-establishment of immune microenvironmental homeostasis. Collectively, this interplay underlies a fundamental ‘restart’ of a multicellular regenerative network. In their role as post-transcriptional regulators, micro-RNAs (miRNAs) exert network-level control over cell fate transitions, paracrine signaling, inflammatory thresholds, and fibrotic processes via the RNA-induced silencing complex (RISC). For this reason, miRNAs have emerged in recent years as key targets for hair regenerative therapies. Recent studies indicate that miRNAs enhance HFSC entry into the active growth phase (i.e., anagen) through pro-regenerative signaling pathways such as Wnt/β-catenin and Sonic hedgehog (Shh), while also mediating the initiation of catagen phase via TGF-β/BMP signaling, extracellular matrix remodeling, and androgen receptor (AR)–associated regressive signaling. In some immune-mediated alopecias (e.g., alopecia areata), miRNA regulation of inflammatory axes, including IFN-γ/JAK–STAT and NF-κB, provides additional therapeutic targets that could potentially be exploited to restore hair follicle immune privilege. In this context, miRNA mimics and inhibitors (e.g., antagomirs and locked nucleic acids, LNAs) enable gain-of-function or blockade, respectively, providing tools that can be used individually or as combined regimens to stimulate regeneration simultaneous with anti-regression/immune remodeling. Recent developments using chemical modifications (e.g., 2′-O modifications, 2′-F, and LNA) improve nucleic-acid stability and modulate RISC loading. From a delivery standpoint, lipid nanoparticles, polymeric nanocarriers, exosome mimetics, and physical enhancement strategies such as microneedles and iontophoresis may help overcome the skin barrier and exploit the follicular “reservoir effect” for localized targeting. Major barriers to clinical translation remain, however, including off-target and network side effects arising from miRNAs’ multi-cellular roles *in vivo*, risk of local immunogenicity and inflammation, alignment of dose–schedule with the hair cycle, and batch consistency with CMC/quality-control requirements for delivery systems. This review summarizes recent developments in understanding the mechanisms, delivery platforms, and translational challenges of miRNAs and their mimics in hair regeneration. It further proposes a translational roadmap centered on modular pathway–guided combinatorial nucleic-acid strategies, follicle-targeted delivery, and standardization of efficacy endpoints to inform future clinical development in this area.

## 1 Introduction

Hair loss disorders are a broad encompassing clinical term generally used to describe conditions such as androgenetic alopecia (AGA), alopecia areata (AA), chemotherapy-/radiotherapy-associated alopecia, and scarring alopecias. These conditions are highly prevalent, often chronic and relapse-prone, and can substantially impair appearance, psychological wellbeing, and quality of life (Davis and Callender, 2018). Although commonly used treatments such as minoxidil, finasteride, corticosteroids, and Janus kinase (JAK) inhibitors can be effective in select patients, clinical management is constrained by variability in efficacy, delayed onset of action, rebound after discontinuation, systemic adverse effects, and/or limited indications. These limitations collectively illustrate that hair regeneration cannot be exclusively controlled by a single pathway and/or mechanism. Rather, it is the result of a “networked regenerative process” jointly constrained by the hair cycle, cellular crosstalk via autocrine/paracrine factors, and the immune microenvironment (Martinez-Lopez et al., 2020). For these reasons, the development of new therapeutic strategies capable of multi-pathway modulation, combined with reducing systemic risk through localized administration, has become a major focus area of research in the field (Jin and Sung, 2024).

At the core of hair regeneration lies the initiation of anagen phase, where hair follicle stem cells (HFSCs) are awakened from quiescence and, under inductive cues provided by the dermal papilla (DP), commit to either proliferation or differentiation, depending on the cues (Yan et al., 2019). Subsequent signals emanating from local immune cells and inflammatory cyto/chemokines ultimately determine whether regeneration can be sustained (Rahmani et al., 2020). These processes are known to be orchestrated by the coordinated interplay between multiple signaling pathways, including Wnt/β-catenin, Sonic hedgehog (Shh), TGF-β/BMP, FGF/IGF, JAK/STAT, and NF-κ (Andl and Botchkareva, 2015). Through their association with the RNA-induced silencing complex (RISC), miRNAs promote target mRNA degradation and/or translational repression, thereby modulating signal integration at the network level in which a single miRNA can regulate multiple targets across multiple pathways. These features make miRNAs intrinsically well suited to hair follicle regeneration as a “multi-node coupled system.” Dynamic changes in miRNA expression have been documented in HFSC activation, maintenance of DP inductive competence, inflammation, and extracellular matrix remodeling. Moreover, it has been shown that miRNAs assist in rebuilding follicular microenvironment via exosome-mediated intercellular communication, positioning them not only as candidate biomarkers of hair regeneration, but also as therapeutic targets (Yan et al., 2019; Andl and Botchkareva, 2015).

From a therapeutic standpoint, miRNA mimics enhance pro-regenerative networks by replenishing or amplifying endogenous miRNA activity, effectively providing a “gain-of-function” modulation. In contrast, miRNA inhibitors (e.g., antagomirs and LNA-modified antisense oligonucleotides, LNA-ASOs) block pro-regressive or pro-inflammatory miRNAs, thereby achieving a “de-repression/noise-reduction” effect (Li and Rana, 2014). Compared with conventional small molecules or single-target biologics, miRNA-based therapies offer two notable advantages. First, they enable combination based targeting strategies for multiple pathway manipulation. For example, in AGA, reinforcement of Wnt/Shh signaling can be coupled with suppression of androgen receptor (AR)–associated regressive cues. In AA, immune homeostasis can be promoted by reprogramming the IFN-γ/JAK–STAT and NF-κB axes, with restoration of follicular immune privilege as a key phenotypic endpoint (Martinez-Lopez et al., 2020). Second, miRNA-based therapies are also well suited for localized delivery, leveraging the cutaneous or follicular “reservoir effect” to achieve high local exposure while reducing the likelihood of adverse systemic effects (Gu et al., 2022). Despite these advantages, the multi-cellular and integrated nature of miRNA activity *in vivo* also increases the risk of off-target effects and network-level perturbations. In addition, challenges related to the skin barrier, cellular uptake efficiency, local immune activation, alignment of dosing schedule with the hair cycle, and the scalability and quality control/chemistry, manufacturing, and controls (CMC) of delivery systems remain critical barriers for translating potential miRNA-based therapies from experimental models into the clinic (Li and Rana, 2014; Gu et al., 2022).

Beyond hair follicle biology, miRNAs have been increasingly recognized as important regulators across a wide spectrum of dermatological conditions. Dysregulated miRNA networks have been implicated in inflammatory skin diseases, autoimmune dermatoses, impaired wound healing, fibrosis, and cutaneous neoplasms, where they modulate keratinocyte differentiation, immune activation, extracellular matrix remodeling, angiogenesis, and epithelial–mesenchymal communication (Sil and Chakraborty, 2024). These observations suggest that miRNAs are not only disease-associated biomarkers but also potential therapeutic modulators in skin biology. Therefore, the investigation of miRNA-based strategies in hair regeneration can be viewed as part of a broader dermatological paradigm in which small non-coding RNAs are used to recalibrate pathological or regenerative tissue programs.

Within this current state of the field, the present review is constructed with the central theme of “mechanistic networks–delivery platforms–translational challenges.” We first describe known pathways through which miRNAs regulate hair regeneration, with emphasis on representative miRNAs and evidence chains across key nodes including HFSC activation, DP inductive competence, regression and fibrosis. Highlighted within this section will be the AR-linked axis in AGA, and the immune axis in AA. We then systematically summarize chemical modification strategies and delivery approaches for miRNA mimics and inhibitors, highlighting the translational potential of nanocarriers, exosome-mimetic systems, and microneedle-enabled local delivery for hair follicle targeting. We then focus on major translational barriers including off-target safety, immunogenicity risk, dose optimization, endpoint evaluation, and CMC standardization. Finally, we will propose (i) mechanism-guided combination regimens centered on miRNA mimics/anti-miRs, and (ii) a development roadmap prioritizing follicle-targeted delivery and standardized clinical endpoints. The ultimate goal of this review is to provide an actionable framework to support future research and clinical translation in hair regeneration.

## 2 Biological foundations of hair regeneration and the therapeutic window

Hair regeneration is not a simple process of hair follicle cell growth and proliferation, but rather a systems-level regenerative program jointly determined by the hair-cycle machinery, the activation threshold of stem cells, the strength of inductive cues, and the immune–stromal microenvironment. Conceptualizing hair regeneration within this framework helps explain two key points: First, miRNAs are more likely to function as network-level tuning modulators, shifting regenerative thresholds and attenuating system noise through coordinated, multi-target regulation. Second, whether miRNA mimics/inhibitors can achieve reproducible efficacy often hinges on the fulfillment of three conditions: appropriate intervention timing (i.e., a defined therapeutic window), precise engagement of target cell populations, and sufficient delivery depth to reach the relevant follicular compartments.

### 2.1 Hair follicle cycling and the anagen entry threshold

The hair follicle cycle comprises anagen (growth), catagen (regression), and telogen (rest) phases, with transitions driven by intracellular molecular clocks and extracellular microenvironmental cues (Lee and Tumbar, 2012). Anagen initiation is not a linear, gradual process. Rather, it more closely follows a threshold-triggered model. It is only when pro-regenerative signals (e.g., Wnt/β-catenin, Shh, and FGF/IGF) accumulate to a level sufficient to override inhibitory inputs (e.g., BMP, TGF-β, DKK1, and inflammatory mediators) that quiescent HFSCs and the secondary hair germ synchronously activated, entering modes of rapid proliferation and tissue remodeling (Plikus et al., 2008; Wang et al., 2023). This gives rise to the so-called “anagen entry window.” This window is the timeframe during late telogen and early transition into anagen, when follicles are maximally responsive to pro-regenerative stimulation. In contrast, during catagen or a heightened inflammatory state, extracellular stimuli may fail to surmount the activation threshold (Plikus et al., 2008).

Hair-cycle dynamics differ widely across species and anatomical sites, presenting challenges for experimental modeling. Animal models—particularly rodents—often exhibit a degree of cycle synchrony, whereas adult human scalp follicles typically cycle asynchronously. Synchronicity of cycle can substantially affect dosing, sampling strategies, and interpretation of efficacy outcomes (Wang et al., 2023). For miRNA-based interventions, this implies that the same miRNA mimic may yield effects that are highly contingent on administration timing and the follicular stage at treatment. Accordingly, study designs should explicitly define the hair-cycle context of the model organism or patient cohort, and “cycle phase” should be treated as a key confounder during analysis and interpretation (Mardaryev et al., 2010).

### 2.2 The engine of hair regeneration: HFSC–DP crosstalk and transit-amplifying cell expansion

The major driving force or “engine” of hair regeneration can be conceptualized as the HFSC–DP axis. HFSCs residing in the bulge region provide the cellular source for regeneration, while the DP, as the mesenchymal inductive center, determines whether regeneration is initiated and governs its quality through paracrine factors, extracellular matrix (ECM) signals, and biomechanical/metabolic cues (Myung and Ito, 2012; Zhang et al., 2024). At anagen onset, HFSCs are activated and give rise to a population of transit-amplifying (TA) cells which undergo rapid expansion and differentiation to form a new hair shaft and inner root sheath (IRS) (Zhang et al., 2024; Hsu et al., 2014). TA cells are responsible for dictating whether hair grows, and also its mass and thickness, serving as a critical amplifier that translates molecular effects into phenotypic outcomes (Hsu et al., 2014).

The DP sustains the directionality and persistence of follicular regeneration by orchestrating pathways such as Wnt, Shh, and FGF/IGF. Conversely, DP aging or loss of inductive competence leads to shortened anagen, thinning of the hair shaft, and follicular miniaturization—histopathological hallmarks that underlie diseases such AGA (Zhang et al., 2024; Inui and Itami, 2011). From a therapeutical perspective, the value of miRNAs within this interactive system lies in their dual leverage: they can regulate the quiescence-to-activation switch in HFSCs, and they can also modulate DP secretion, cellular senescence, and paracrine communication (Yang et al., 2021).

### 2.3 Microenvironmental limits: immune privilege and inflammatory thresholds

Hair follicles are classic examples of immune privilege structures *in vivo*, manifesting as low antigen presentation, local release of immune suppressive factors, and the coordination of regulatory immune cell populations (Yan et al., 2019). When immune privilege is disrupted, the infiltration of inflammatory cyto/chemokines and effector T Cells/cytotoxic lymphocytes (CTLs) can drive hair follicles into catagen or suppress HFSC function as seen in AA (Bertolini et al., 2020; Xing et al., 2014). In AGA, chronic low-grade inflammation and oxidative stress can also alter the signaling balance between the DP and epithelial cells, thereby elevating the anagen initiation threshold (Mahé et al., 2000; Upton et al., 2015). Consequently, hair regeneration operates within an upper limit defined by immune status. When the inflammatory threshold is raised, simply enhancing pro-regenerative pathways is often insufficient to reverse the cycle. Simultaneously reducing inflammatory noise and promoting the restoration of immune privilege are essential parameters for enabling regenerative signals to cross the threshold (Bertolini et al., 2020; Xing et al., 2014).

This also explains the unique potential of miRNAs from a therapeutic exploitation standpoint. Certain miRNAs can simultaneously target both regenerative and inflammatory signaling nodes (e.g., modulating NF-κB or JAK/STAT pathways), thereby aligning with the systemic needs of the follicular microenvironment. Such a strategy could be considered a dual pro-regeneration + immune remodeling approach. However, it is crucial to emphasize that given the immuno-modulatory properties of miRNAs, safety assessments should not be limited to hair growth metrics alone. They should also consider local inflammation, barrier function, and changes in immune cell profiles to avoid risks such as triggering or exacerbating inflammation.

### 2.4 Efficacy assessment

Hair regeneration operates on a time scale of weeks to months, significantly influenced by the follicular cycle phases and microenvironmental thresholds. For this reason, the dosage, frequency, and duration of nucleic acid-based therapies must align with the hair follicle cycle. Too short a treatment duration may only alter molecular markers without affecting visible hair shaft changes. Conversely, excessive or too frequent dosing may counteract any beneficial effect due to local immune activation or cellular stress (Jin and Sung, 2024). Compounding these challenges is that the skin barrier makes effective delivery more complex than mere topical application. To yield effective hair regeneration, drugs must spatially reach the follicular unit (especially the bulge and DP regions) and temporally coincide with the anagen entry window (Jin and Sung, 2024; Lademann et al., 2006).

Thus, a translatable miRNA mimic strategy must at the very least meet a four-dimensional evidence chain simultaneously: ① Spatial evidence (e.g., target follicle/cell localization and uptake); ② Mechanistic evidence (e.g., RISC loading, strand selectivity, and expected changes in target genes/pathways); ③ Phenotypic evidence (e.g., progression of the hair follicle cycle, improvements in hair density/diameter/terminal hair ratio, and clear onset of efficacy); ④ Safety evidence (e.g., minimal signs of local irritation, inflammation, pigment changes, and reversibility).

## 3 Mechanistic network

**FIGURE 1:**
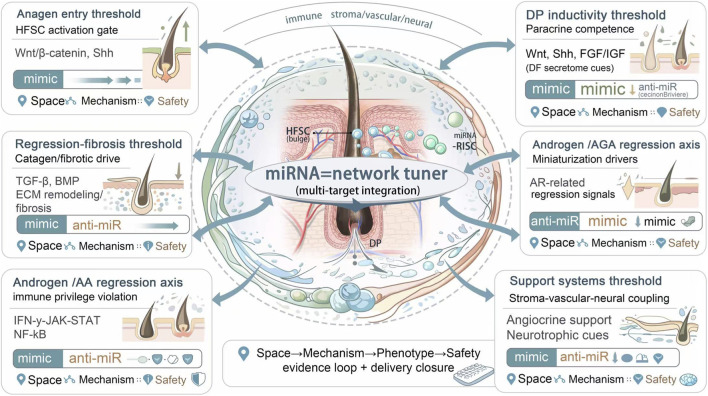
The Mechanistic Module Map of miRNA Regulation in Hair Regeneration.

Hair regeneration is not a physiological process driven by a single linear pathway, rather it is a branched network comprised of HFSCs, DPs, immune cells, and the stroma/vascular–neural components, all of which are collectively re-activated at specific phases (Ji et al., 2021). Within this system, miRNAs serve multi-faceted roles, where a single miRNA can simultaneously affect pro-regenerative and anti-regenerative pathways. In this way, they are capable of modulating key thresholds such as activation, regression, and inflammation in a network-level manner. The most insightful and translatable miRNA pathways identified to date that are relevant in this context can be summarized into six mechanistic modules (Figure 1).

### 3.1 HFSC activation and anagen initiation: Wnt/β-catenin module

Wnt/β-catenin is widely regarded as the “gatekeeper” for anagen entry. Its activation promotes HFSCs to transition from quiescence to proliferation and differentiation, whereas its inhibition keeps the hair follicle in a high-threshold state (Myung et al., 2013). The typical role of miRNAs in this module is to relieve Wnt inhibition or enhance β-catenin transcriptional activity (Table 1), thereby lowering the anagen entry threshold. Other studies suggest miRNAs may target multiple inhibitory nodes such as extracellular antagonists (e.g., DKK1/SFRPs) or intracellular negative regulators (e.g., GSK3β/APC/CTNNBIP1), thereby altering β-catenin stability, nuclear translocation, and downstream transcription (Enshell-Seijffers et al., 2010; Zhao et al., 2019).

**TABLE 1:** miRNA candidates in the Wnt/β-Catenin module: key targets, primary effector cells, evidence level, and major readouts.

miRNA (direction)	Key target/Node (relation to wnt gatekeeping)	Primary effector cells/Region (location-dependent)	Evidence level & model	Major readout	Representative literature
miR-218–5p (↑Wnt, promotes anagen)	SFRP2↓ (extracellular Wnt antagonist) → β-catenin signal enhancement	DP → Epithelium (Exosomal miRNA from DP enters follicular epithelium/HFSC region)	A/C: Mouse dermal/subcutaneous injection; DP exosome; miR-218–5p mimic/inhibitor intervention	SFRP2 downregulation, β-catenin upregulation, hair growth/coverage, anagen initiation	Hu et al. (2020a)
miR-214 (↓Wnt, inhibits growth/delays anagen)	β-catenin↓ (direct targeting) → Wnt-mediated transcription (e.g., LEF1) reduction	Epidermal progenitor cells/ORS (HFSC-related epithelium)	A/C: Keratinocyte-specific overexpression in mice; pharmacological Wnt activation can rescue phenotype	HF number, hair bulb size, hair shaft thinning; β-catenin, LEF1 downregulation; Wnt activator rescue	Myung et al. (2013)
miR-195–5p (↓Wnt, reduces DP inductivity)	Predicted function: LRP6↓ (Wnt co-receptor) → β-catenin signal attenuation	DP cells (affects “inductive capacity and regenerative quality”)	B/C: Human scalp samples + DP cell *in vitro*; miR-195–5p overexpression/inhibition	β-catenin/LEF1/Wnt3a downregulation; DP inductivity-related markers (e.g., ALP/VCAN/FGF7) reduced	Enshell-Seijffers et al. (2010)
miR-29a/b1 (↓Wnt, inhibits HFSC lineage progression/causes hair loss)	LRP6, CTNNB1 (β-catenin), etc. (parallel BMP axis: BMPR1a, etc.) → inhibits Wnt/β-catenin and HFSC progression	HFSC/Epithelial lineage (more sensitive on the gatekeeping side for entering anagen)	A/C: Mouse + cell; overexpression leads to hair loss phenotype	HF lineage progression blocked; Wnt/BMP pathway readout decreased; follicle/hair phenotype altered	Myung et al. (2013)
miR-24 (↓Wnt output, tends to suppress epithelial stemness/affect morphogenesis)	TCF-3↓ (direct targeting): TCF/LEF family members, at the β-catenin nuclear transcription output	Hair follicle epithelium/Keratocyte lineage	A/C: Transgenic/*in vivo* intervention + *in vitro* validation (literature review summary)	HF morphogenesis defects, hair thinning; TCF-3 downregulation; proliferation and differentiation marker changes	Enshell-Seijffers et al. (2010)
miR-31 (maintains Wnt-BMP-FGF balance, generally promotes normal growth)	Multi-target balanced regulation (review/originally described as regulating key Wnt/BMP/FGF components)	Hair follicle (high expression during anagen)	B/C: Skin/hair follicle gene program-level evidence; more of a network homeostasis regulator	Hair follicle cycle-related gene program; tissue remodeling; pathway balance readout (Wnt/BMP/FGF)	Zhao et al. (2019)
miR-148 b (↑Wnt, promotes proliferation/regeneration candidate)	NFAT5↓ → Wnt10b/β-catenin axis↑ (indirect enhancement of Wnt/β-catenin)	Hair follicle cells (described as HF-related cells)	C: Primarily *in vitro* functional validation (literature summary)	Wnt10b/β-catenin upregulation; increased cell proliferation	Hu et al. (2020a)
miR-133 b (↓Wnt, AGA-related negative candidate)	Mechanism described as Wnt/β-catenin inactivation (specific targets need to be clarified in original studies)	AGA scalp-related cells (typically DP/hair follicle microenvironment)	B/C: AGA samples + *in vitro*	β-catenin/versican, etc., readouts down; hair growth-related indicators worsened	Zhao et al. (2019)
miR-329 (↓Wnt, DP maintenance/regeneration axis candidate)	Wnt10 b axis (suggested by upstream lncRNA-PCAT1/miR-329/Wnt10 b regulation chain)	DP cells	D→B (depending on whether you expand on this lncRNA axis in the text): Sample/mouse/cell axis evidence	DP characteristics maintained, Wnt10 b changes, regeneration phenotype	Enshell-Seijffers et al. (2010)
miR-130b-3p (↓Wnt, development/species evidence)	Wnt10a↓ (reported target) → inhibits Wnt/β-catenin	Skin epithelium/fibroblast-related cells (mainly studied in livestock hair follicle development)	D/C: Mainly found in livestock skin/hair follicle development *in vitro* systems	Cell proliferation, Wnt10a and β-catenin axis readout	Myung et al. (2013)

It is important to emphasize that the regulatory strength of the Wnt module in hair regeneration is highly dependent on location of the target cells. Wnt activation in HFSCs directly influences whether these cells enter anagen, whereas Wnt activation in DPs enhances inductive capacity and regenerative quality (Myung et al., 2013; Enshell-Seijffers et al., 2010). Recent studies provide further physiological relevance to this module where it was shown that exosomal miRNAs derived from the dermis/DP can enter epithelial cells and enhance β-catenin signaling. This suggests that indirect DP-driven HFSC activation via exosomal miRNA is feasible (Hu S. et al., 2020).

Pharmacological Potential Review: Based on its role as summarized above, the Wnt module is more suitable for precise, localized, and temporal window-based interventions. Excessive Wnt activation may lead to abnormal proliferation or non-specific reactions in skin appendages (Gat et al., 1998). Therefore, the recommended strategy should involve moderate Wnt enhancement and concurrent anti-inflammatory/anti-regression signaling, using follicular-targeted delivery to minimize widespread epidermal exposure. At the same time, spatial targeting evidence for bulge/HFSC or hair bulb/DP-related regions should be sought after to avoid false negatives (e.g., signal upregulation without key cellular targeting).

### 3.2 Importance of the paracrine and exosomal module for maintaining DP inductive capacity and youthfulness

**FIGURE 2:**
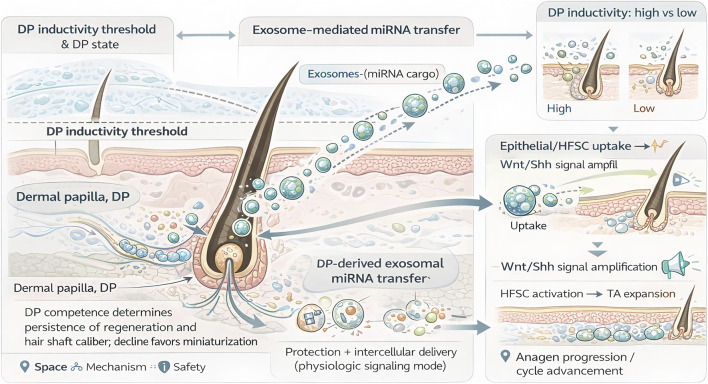
Regenerative Communication Mediated by Dermal Papilla-Derived Exosomal miRNAs: DP/Dermis–Epithelial/HFSC Axis and Signal Amplification.

The DP is the inductive center for hair follicle regeneration, and its capacity determines whether regeneration can be sustained, the thickness of the hair shaft, and whether follicular miniaturization can be reversed (Enshell-Seijffers et al., 2010). From a network perspective, this module primarily regulates the “DP inductive threshold,” where the DP can output sufficient pro-regenerative signaling and the correct secretory profile to ignite and maintain HFSC–TA amplification. DP functional decline is often manifested by altered secretory profiles, increased cellular senescence, oxidative stress, and insufficient output of pro-growth signals to epithelial and stem cells (Upton et al., 2015). Studies involving miRNAs within this module mainly focus on two aspects (Figure 2). The first aspect involves endogenous miRNA regulation of DP phenotype and secretory profile. Some miRNAs can indirectly maintain the DP “youthfulness” by affecting cell cycle, mitochondrial stress, or senescence-related pathways, thereby enhancing its inductive capacity toward HFSCs (Zhu et al., 2018). The second aspect involves DP-derived exosomal miRNAs as the vehicles, or carriers, of intercellular communication. Exosomes not only provide inherent protection of the miRNA contents but also possess intercellular transfer properties, making miRNA transmission more akin to real physiological signaling. Once exosomal miRNAs enter epithelial cells, they can amplify signaling in pro-regenerative pathways such as Wnt and Shh, thereby promoting the progression of the hair follicle cycle (Chen et al., 2020).

Pharmacological Potential Review: There are unique advantages of the exosomal pathway in terms of biocompatibility and signal biomimicry, but there are challenges involving standardization and controllability (e.g., source cells, loading efficiency, batch consistency) (Ahn et al., 2022). In terms of translation, the DP exosome mechanism can be used as the biological rationale, while future developments in formulation should prioritize controllable and scalable biomimetic carriers (i.e., nanocarriers) to replicate exosomal delivery. Formulations should specifically focus on follicular targeting, cellular uptake, and low immunogenicity.

### 3.3 Regression and fibrotic tendencies: TGF-β/BMP/ECM module

The initiation of catagen (regression) and tissue remodeling is driven by the TGF-β/BMP axis, apoptosis signaling, and ECM remodeling (Foitzik et al., 2000). BMP is typically associated with the maintenance of HFSC quiescence (Plikus et al., 2008), while TGF-β signaling plays a crucial role in initiating catagen, amplifying inflammation, and promoting fibrosis. The typical role of miRNAs in this module involves suppression of TGF-β/Smad signaling to reduce the regression–fibrosis threshold, or moderate modulation of BMP signaling to alleviate excessive quiescence. Concurrently, some miRNAs can affect the expression of ECM-related genes and fibroblasts, thereby influencing the regenerative microenvironment’s progression from reversible remodeling to a scarring phenotype (Xu et al., 2021).

The most important feature of this module is the “double-edged sword effect”. Specifically, while inhibition of fibrosis and ECM deposition favors the restoration of the regenerative microenvironment, excessive TGF-β/BMP signaling could affect normal tissue repair and immune balance. A main barrier here is that the same miRNA can have different target profiles in different cell types (e.g., epithelial, fibroblasts, immune cells), potentially leading to contradictory phenotypes. Therefore, miRNA related studies in this area should emphasize not simply whether hair growth phenotypes are observed, but whether the miRNA truly reduces regression, improves ECM remodeling, and maintains barrier and immune safety.

Pharmacological Potential Review: This module is more suited for the use of anti-miRs or combinatorial strategies (e.g., Wnt enhancement combined with regression/fibrosis inhibition), with delivery strategies focused on the follicular unit and superficial dermis to avoid non-specific inhibition across the entire skin layer.

### 3.4 AR–DKK1–TGF-β and oxidative stress/inflammation coupling module: AGA key axis

**FIGURE 3:**
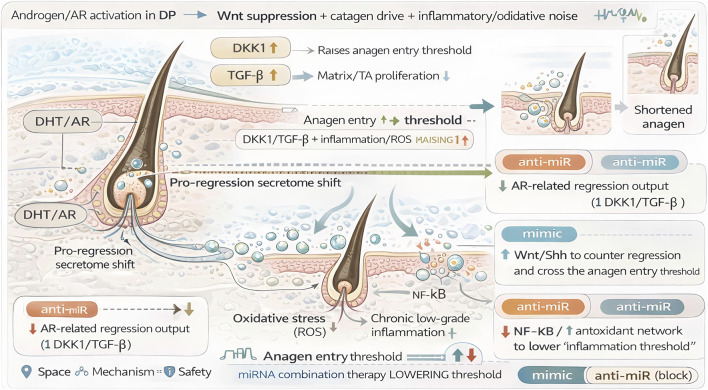
Key Pathway Axis in AGA: AR–DKK1/TGF-β Mediated Wnt Inhibition, Follicular Miniaturization, and miRNA Intervention Points.

One of the core pathologies of AGA is the androgen-driven alteration of the DP microenvironment (Figure 3). DHT/AR signaling can skew the DP secretory profile toward regression promotion, typically manifested as enhanced Wnt inhibition (e.g., upregulation of DKK1) and increased TGF-β–related regressive signals. This is accompanied by low-grade inflammation and oxidative stress, ultimately leading to follicular miniaturization and shortened anagen (Hsu et al., 2014; Xing et al., 2014; Mahé et al., 2000; Xu et al., 2021). Therefore, from a threshold perspective, the essence of AGA pathophysiology is the combined effect of regression-driven signals and inflammatory noise, which elevates the activation threshold and shortens anagen. Studies in this area have shown that miRNAs are better suited for reducing regression output, reducing inflammatory noise and moderately enhancing pro-regenerative signals to reshape the threshold balance (Inui and Itami, 2011; Mahé et al., 2000; Upton et al., 2015).

Mechanisms of miRNA action in AGA can be summarized into three pathways: 1) Inhibiting AR-related regressive signals by regulating key nodes in the AR signaling pathway or its downstream pro-regressive factors, reducing the intensity of inhibitory signals such as DKK1/TGF-β (Inui and Itami, 2011; Kwack et al., 2008). 2) Enhancing pro-regenerative pathways to counter regression by strengthening pathways such as Wnt/Shh in DPs or HFSCs, to push the system past the anagen initiation threshold (Inui and Itami, 2011). 3) Relieving oxidative stress and chronic inflammation by disrupting NF-κB-related negative feedback or enhancing antioxidant networks, to reduce the elevating impact of inflammatory thresholds on anagen initiation (Mahé et al., 2000; Upton et al., 2015).

Pharmacological Potential Review: From a standpoint of therapeutic strategy, AGA aligns more with a rationale of combination therapies. Simple upregulation of a single pro-growth pathway is often insufficient for long-term effects, and a therapy must simultaneously address regression drivers and background inflammation (Inui and Itami, 2011; Mahé et al., 2000). AGA patients require long-term localized management, so miRNA mimics need to consider long-term safety and controlled dosing frequency. For clinical translation, priority should be given to candidate miRNAs with relatively focused target profiles that exhibit minimal off-target effects in non-skin tissues. They should also retain the ability to be coupled with microneedle or follicular reservoir delivery systems.

### 3.5 Breakdown of immune privilege and the IFN-γ/JAK-STAT module: AA key axis

**FIGURE 4:**
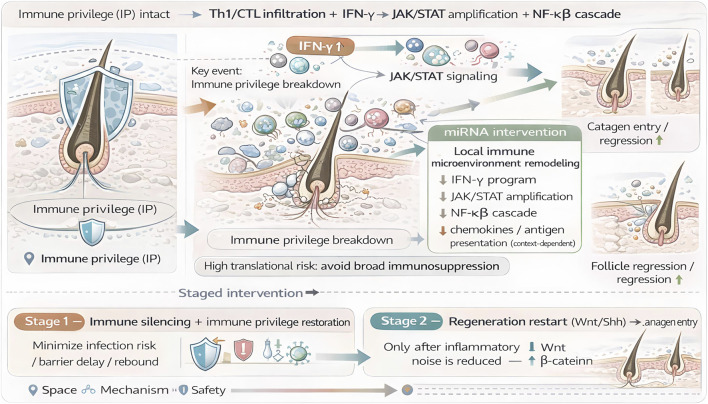
Immune gate failure model in AA: Breakdown of immune privilege driving IFN-γ/JAK-STAT amplification and follicular regression with temporal intervention I.nterface.

The critical issue in pathophysiology of AA is the disruption of hair follicle immune privilege (Figure 4). Here, enhanced Th1/CTL, IFN-γ, and JAK/STAT signaling lead to continuous inflammatory stress on hair follicle epithelial cells, driving the follicle into either catagen or regeneration failure (Bertolini et al., 2020; Xing et al., 2014). The significance of miRNAs in potential treatment of AA lies in their ability to directly regulate key nodes of inflammation (e.g., IFN-γ, JAK/STAT, and NF-κB-mediated gene expression), or to alter antigen presentation, cyto/chemokines, and immune cell differentiation, thereby changing the intensity and profile of immune cell infiltration (Wang et al., 2017).

However, the translational requirements for treating AA are more stringent. Improper alteration of inflammatory and immune responses can lead to local infection risks, delayed barrier repair, or immune rebound (Papierzewska et al., 2023). For this reason, miRNA interventions in AA are better suited for local immune microenvironment remodeling rather than broad immune suppression. A more robust approach would be to place miRNA strategies within a framework of immune silencing and regenerative restart. This could be accomplished by first reducing background inflammation and restoring immune privilege, then utilizing Wnt/Shh and other mediators to nudge hair follicles into anagen, avoiding any situations where pro-growth signals could become engulfed by background inflammation.”

Pharmacological Potential Review: Localized targeted delivery is the preferred strategy in AA treatment, with a focus on reversible, drug-withdrawal-capable delivery systems. The development strategy should center around minimizing the uncertainty of immune rebound and infection risks. Additionally, immune-related endpoints (e.g., infiltration cell profiles, IFN-γ axis markers, and barrier and microbial risk assessments) should all be evaluated to meet safety and mechanistic consistency requirements.

### 3.6 Vascular-neural-inflammatory interplay module

Hair follicle regeneration relies on metabolic and nutritional support, where the vascular and nervous systems not only provide blood supply and sensory regulation but also immune modulation and anagen maintenance *via* neuropeptides and cyto/chemokines. Angiogenesis (e.g., VEGF) has been shown to improve local oxygenation and nutrition, thereby supporting anagen maintenance (Yano et al., 2001; Li et al., 2012). Neural-related signals are often linked with immune status and may also influence the hair follicle’s sensitivity to inflammation (Peng et al., 2022; Peters et al., 2007). Potential therapeutic value of miRNAs in this module lie in their ability to regulate angiogenesis, hypoxic responses, and expression of neuro-immune mediators, providing a more stable external environment for the HFSC–DP axis to support regeneration (Mardaryev et al., 2010; Sen and Roy, 2012).

Pharmacological Potential Review: This module may be suitable as a supplementary component or part of a combined strategy but is unlikely to be effective as a standalone primary target. One rational approach could be in combination with Wnt/immune modulators to enhance regeneration sustainability and quality. Since this vascular-neural-inflammatory module primarily regulates regenerative support (e.g., blood supply, oxygenation, metabolism, and neuro-immune coupling), it will be more effective at influencing anagen maintenance and regeneration persistence rather than triggering initiation.

## 4 Chemical modifications, pharmacological mechanisms, and delivery strategies of miRNA mimics

The underlying rationale behind miRNA mimics is to translate the functional characteristics and actions of endogenous miRNAs into an actionable therapeutic, and it remains of the most programmable strategies in hair regeneration research (van Rooij and Kauppinen, 2014; Rupaimoole and Slack, 2017). However, unlike small molecules, the efficacy of miRNA mimics does not solely depend on tissue delivery but rather on whether they can be properly loaded onto the RISC in the correct cells and exert sufficient target gene suppression with adequate intensity and duration (Setten et al., 2019).

Therefore, this chapter reviews key aspects of miRNA mimics ranging from molecule to translatable therapy, covering pharmacological mechanisms, chemical modifications, and delivery strategies. It also covers strategies for delivery systems and current barriers for clinical implementation.

### 4.1 Mechanism of action of miRNA mimics

miRNA mimics are typically composed of double-stranded RNA, where the “guide strand” mimics the mature miRNA sequence and is loaded onto the RISC after cellular uptake, and the “passenger strand,” which initially is there for stability, becomes degraded (Schwarz et al., 2003). Once loading is complete, the RISC-guide strand pairs with the target mRNA through the seed region (typically positions 2–8) to induce complementary base pairing, often in the 3′UTR, although base pairing can occur in other regions (e.g., CDS) (Bartel, 2009). This interaction represses mRNA translation and post-translational processes such as adenylation removal and de-capping, leading to decreased mRNA stability and reduced protein expression (Jonas and Izaurralde, 2015; Stenn and Paus, 2001).

For hair regeneration, the key to miRNA mimic action is not maximal inhibition of any particular pathway, but rather achieving a threshold-level network reorganization. One example of this reorganization could involve lowering the threshold for anagen entry in HFSCs, enhancing induction signal strength in DPs, and reducing inflammation or restoring immune privilege (Bartel, 2009).

### 4.2 Chemical modification of miRNA mimics and their impact

Exposed RNA is prone to nuclease-mediated degradation in bodily fluids and tissues, and double-stranded RNA may trigger innate immune responses. Therefore, miRNA mimics typically require chemical modifications to balance stability, activity, and safety (Hu B. et al., 2020). Common modifications can be categorized into three main categories described as follows, and listed in Table 2:

**TABLE 2:** Common chemical modifications in miRNA Mimics: stability, RISC loading/strand selectivity, immunogenicity, and potential trade-offs.

Chemical modification	Primary effect	Modification details	Impact on delivery/Targeting effect	Immunogenicity/Safety impact	Representative literature
2′-O-Methylation (2′-OMe)	Enhance stability and nuclease resistance	Methyl group (CH3) modification at the 2′-position of the nucleotide, improving resistance to degradation and extending tissue half-life	Increases stability, extends the half-life of the delivery system in blood and tissues	Low: Moderately reduces immune activation, but high concentrations may cause non-specific binding	Deleavey and Damha (2012)
2′-O-Fluorination (2′-OF)	Enhance stability and nuclease resistance	Fluorine (F) modification at the 2′-position of the nucleotide, significantly improving resistance to degradation	Significantly enhances stability, increasing the retention time of the miRNA mimic *in vivo*	Low: Compared to other modifications, it has lower immunogenicity and toxicity	Crooke et al. (2020)
Phosphorothioate Modification (PS)	Enhance stability and nuclease resistance	Introduction of a sulfur atom (P-S bond) into the phosphate backbone, enhancing stability and binding capacity	Enhances stability and increases protein binding affinity	Moderate: Requires control of ratio; excessive levels may cause non-specific binding and toxicity	Bramsen et al. (2009)
Terminal Asymmetry Design	Optimize RISC loading and strand selectivity	Asymmetric modification at the ends of the passenger strand (e.g., different terminal chemical groups)	Optimizes strand selectivity, prevents misloading of the passenger strand, and reduces non-specific silencing	Low: Reduces off-target effects by minimizing misloading	Bramsen et al. (2009), Khvorova et al. (2003)
Passenger Strand Modification	Optimize RISC loading and strand selectivity	Modifications such as 2′-O-methylation or 2′-fluorination on the passenger strand to reduce its loading into RISC	Reduces misloading, enhances guide strand loading efficiency	Low: Ensures precise strand selectivity, preventing unintended target suppression	Bramsen et al. (2009), Khvorova et al. (2003)
2′-O Modification (Moderate)	Reduce immunogenicity and inflammation risk	2′-O-methylation or 2′-fluorination modifications to enhance stability while reducing immune activation	Reduces immune activation, helping to minimize local inflammation, suitable for topical delivery	Low: Moderate modification reduces immune response, but excessive modification may impair RISC loading efficiency	Robbins et al. (2007)
GU-rich Sequence Optimization	Reduce immunogenicity and inflammation risk	Reduces GU-rich sequences (decreasing TLR3, RIG-I/MDA5 recognition)	Reduces immune activation and avoids unnecessary immune recognition	Low-Moderate: Prevents immune response and lowers local inflammation risk	Judge et al. (2005), Capaldi et al. (2017)

Enhancing Stability and Nuclease Resistance: Modifications at the 2′position (e.g., 2′-O-methyl, 2′-fluoro) can significantly improve resistance to degradation, extending the tissue half-life (Deleavey and Damha, 2012). Phosphorothioate (PS) backbone modifications also enhance stability and protein binding but must be carefully controlled to avoid non-specific binding and toxicity (Crooke et al., 2020).

Optimizing RISC Loading and Strand Selectivity: Mimics need to preferentially load the guide strand onto RISC, so modifications are often introduced on the passenger strand or asymmetry is designed at the ends to reduce the probability of erroneous loading of the passenger strand, thus minimizing non-specific silencing (Bramsen et al., 2009; Khvorova et al., 2003). Strand selectivity is particularly critical for hair regeneration, where network threshold regulation is the objective. If the passenger strand is incorrectly loaded, it could result in unintended target suppression and amplification of the off-target effects (Bramsen et al., 2009; Khvorova et al., 2003).

Reducing Immunogenicity and Inflammatory Risk: Double-stranded RNA can be recognized by inflammatory receptors (e.g., TLR3, RIG-I/MDA5, and others), triggering problematic inflammatory responses, especially when administered locally in the skin. Moderate 2′-O modifications generally help reduce immune activation, but excessive modification may impair RISC loading and target mRNA silencing (Judge et al., 2005; Robbins et al., 2007). Additionally, the immunostimulatory nature of the miRNA sequence itself (e.g., GU-rich regions) and production impurities (e.g., dsRNA contaminants, truncated species) can significantly enhance the risk of inflammation (Judge et al., 2005; Capaldi et al., 2017).

### 4.3 Complementary relationship between anti-miR (Antagomir/LNA-ASO) and mimic

miRNA mimics effectively result in a gain-of-function phenotype, and as such they are more appropriately suited for pro-regenerative modules (e.g., enhancing Wnt/Shh signaling, improving DP inductive capacity). Conversely, anti-miRs/ASOs typically result in a loss-of-function phenotype and are more appropriate for “de-noising” interventions targeting pro-regressive or pro-inflammatory miRNAs that reduce regression and inflammation, respectively (Krützfeldt et al., 2005; Obad et al., 2011; Lindow and Kauppinen, 2012). More importantly, anti-miRs are more likely to exhibit net effects in a “high-threshold background” (e.g., strong inflammation, regression, or fibrotic tendencies) because they first reduce noise and suppress network intensity, thereby freeing up space for pro-regenerative modules (Krützfeldt et al., 2005; Obad et al., 2011).

These two strategies, mimics and anti-miRs, need not be competitive but could even be complementary, corresponding to “accelerators” and “brakes/noise reducers” respectively within the hair regeneration network (Lindow and Kauppinen, 2012). Examples of where complementary strategies would be appropriate are in treating complex diseases like AGA and AA, where mimic treatment alone is often countered by regressive or inflammatory signals. Here, a more favorable strategy is to use mimics to initiate regenerative pathways while employing anti-miRs to lower the regression/inflammation thresholds (Lindow and Kauppinen, 2012).

### 4.4 Aligning drug delivery strategies with “modules” and “therapeutic windows”

Hair follicle regeneration treatments have long evaluation cycles and are highly variable. Therefore, miRNA mimic drug delivery strategies must be designed with the following specific modules and therapeutic windows in mind (Oh et al., 2016).

#### 4.4.1 Choosing the primary target module and target cells

HFSC/Wnt module: The goal here is to lower the anagen entry threshold, emphasizing short duration, therapeutic window coverage, and avoiding excessive stimulation.

DP module: The goal here is to enhance the strength and persistence of inductive signals, potentially using a longer treatment duration while controlling risk of local inflammation.

Immune module (AA): The goal here is to restore immune privilege, with a stronger focus on safety and monitoring immune-related endpoints.

#### 4.4.2 Dosing frequency and duration: prefer “adequate coverage” over “excessive dosing”

Since the hair follicle cycle spans weeks to months, a typical strategy involves observing molecular and early phenotypic changes over several weeks before deciding whether to intensify or extend the treatment. Excessive dosing frequency or concentration may induce local immune activation or non-specific stress, potentially negating growth benefits (Oh et al., 2016). It is recommended to observe patients carefully for signs of drug withdrawal and/or reversibility in the protocol, particularly for conditions requiring long-term management (e.g., AGA).

#### 4.4.3 Prioritizing local delivery and operability

The most practical path for mimics in the field of hair regeneration is local administration, using follicular reservoirs and physical enhancements (e.g., microneedles) to improve delivery (Lademann et al., 2006; Deng et al., 2016). In other words, the drug delivery strategy cannot exist independently of the delivery system. If delivery is limited to the epidermal layer, mimics will have difficulty acting on HFSCs/DPs. However, if delivery reaches the follicular unit, it becomes easier to achieve low-dose, low-frequency interventions over the long-term (Lademann et al., 2006; Deng et al., 2016).

## 5 Delivery system

Whether miRNA mimics/inhibitors can truly become a translatable therapy for hair regeneration depends not only on whether the nucleic acid sequence is optimal, but on whether the mimics can cross the skin barrier and reach the key target cells within the hair follicle unit (HFSCs, DP, and associated immune cells) (Elias, 2005), while maintaining sufficient local concentration and duration (Sufianov et al., 2023). Unlike high-uptake organs like the liver, the skin has low natural permeability to nucleic acid-based drugs, is rich in immuno-modulatory receptors, and the follicle target cells are distributed in three-dimensional space. This necessitates that the delivery system follows an engineering-based pathway consisting of molecular modification → carrier encapsulation/complexation → formulation (e.g., gel, patch, topical system, etc.) → skin penetration → follicular targeting/reservoir → intracellular release and pharmacodynamic validation → safety and tolerability evaluation.

### 5.1 Skin barrier and hair follicle “reservoir effect”

The primary physical obstacle to skin delivery is the stratum corneum, whose brick wall-like structure strongly repels hydrophilic, charged macromolecules. Since miRNA molecules themselves are negatively charged and susceptible to nuclease degradation, simple topical application often results in retention only in the superficial epidermis (Sufianov et al., 2023). Additionally, the dermis contains a rich array of innate immune receptors (e.g., TLRs, RIG-I-like receptors), and double-stranded RNA or certain carrier materials may trigger local inflammation. In turn, this may cause erythema, irritation, and barrier damage, which could increase the inflammatory threshold and negate regenerative signals (Kalali et al., 2008).

The hair follicle unit also provides a bypass entry. Specifically, the follicular infundibulum and sebaceous gland area create relatively low-resistance channels, giving rise to the so-called “hair follicle reservoir effect,” where particles or carriers can be retained in the follicle opening and hair follicle duct, releasing slowly over time (Blume-Peytavi and Vogt, 2011). However, the reservoir effect does not equate to effective targeting. The actual therapeutic targets (bulge region HFSCs and DP) are located deeper within the follicle and stay there longer (Lademann et al., 2007). Moreover, there are significant variations in follicle density and associated hair parameters across different scalp regions which may affect the consistency in entry accessibility, reservoir capacity and local exposure (Rutnin et al., 2022). Therefore, hair regeneration delivery systems must be capable of addressing two key issues: (1) they must be able to enter the follicular pathway and form stable retention/controlled release, and (2) they must be able to reach deeper compartments like the bulge/DP and be effectively taken up and released by target cells.

### 5.2 Nanocarriers with emphasis on lipid nanoparticles

In nucleic acid delivery, the primary task of nanocarriers is to shield the charge, protect the nucleic acids from degradation, enhance cellular uptake, and enable effective intracellular release. For skin and hair follicle targeting, nanocarrier design must also consider local tolerance and operability.

1. Lipid Nanoparticles (LNPs)

LNPs are typically comprised of ionizable lipids, auxiliary lipids, cholesterol, and PEG-lipids. These constituents are capable of efficiently encapsulating nucleic acids and promoting endosomal escape, making them a mature and widely used nucleic acid delivery platform. Advantages of LNPs for topical skin application include high encapsulation efficiency, tunable particle size and surface properties, and amenability to scalable manufacturing. However, their limitations include the potential for certain lipid components to induce local irritation and/or inflammation. While PEGylation improves stability, it may reduce cellular uptake, and the immune concerns associated with long-term repeated dosing need to be evaluated (Horejs, 2021). For follicular delivery, LNPs are better suited for combination with physical enhancement methods (e.g., microneedles) to improve penetration depth, rather than relying solely on topical permeation. However, several challenges remain, including limited penetration through the stratum corneum, possible irritation induced by lipid components, PEG-related effects on cellular uptake, local innate immune activation, and the need to evaluate safety under repeated dosing conditions. Therefore, future LNP-based miRNA delivery systems for hair regeneration should be optimized not only for encapsulation efficiency but also for follicular retention, intracellular release, local tolerability, and reproducible manufacturing.

2. Polymeric Nanocarriers

Cationic polymers can form nano-complexes with nucleic acids *via* electrostatic interactions, improving stability and uptake efficiency. Some polymers also facilitate endosomal escape through the “proton sponge effect.” The advantages of polymeric carriers include highly customizable materials, integration with hydrogels/patch systems, and suitability for localized controlled release. However, their limitations include potential cell toxicity and inflammatory responses due to high cationic density, as well as the need for strict validation of batch consistency and the safety of degradation products (Pack et al., 2005). For hair follicle regeneration, it is recommended to use polymers with a “gentle” charge and biodegradable backbone, with a focus on localized, controlled release.

3. Inorganic/Hybrid Nanomaterials

Inorganic materials such as gold nanoparticles, silica, and layered materials can serve as nucleic acid adsorption or loading platforms. These offer advantages of structural stability and surface functionalization, as well as enabling stimulus-responsive release. However, their biocompatibility, metabolic clearance, and potential chronic inflammation risks during long-term skin use limit their priority as a first-line translatable solution (Luther et al., 2020). A more realistic application is to use these nanomaterials as a tool for mechanistic validation or verification of a delivery concept. They also may be applicable for translation after identifying a safe usage window.

Overall, the selection of nanocarriers should prioritize local tolerance, scalability and repeatable dosing. In hair regeneration, the carrier itself cannot replace the challenge of deep hair follicle penetration. Rather, it typically requires synergistic use with physical enhancement or follicle-targeting strategies.

### 5.3 Exosomes and exosome-mimetic vesicles as biomimetic miRNA delivery systems

Exosomes (extracellular vesicles, EVs) are considered highly attractive biomimetic delivery carriers due to their natural ability to carry nucleic acids and proteins and their role in intercellular communication (Kim et al., 2024). In hair regeneration research, exosomes derived from DP or mesenchymal cells have been shown to carry miRNAs and promote regeneration of epithelial cells/HFSCs. This provides direct evidence for biological rationale and integrated delivery, since they serve as both the signaling entity and the delivery carrier (Hu S. et al., 2020).

The potential advantages of exosome delivery include high biocompatibility, lower propensity for immune activation, and a more physiologically relevant distribution in the local microenvironment. Additionally, exosomal membrane proteins and glycosylation features may confer tissue/cell specificity (Kim et al., 2024). However, the main barriers to exosomal implementation are related to their bioengineering and CMC. Variations in source cells significantly affect the composition and function of exosomes, loading efficiency and consistency are difficult to standardize, and issues such as scalable production, purification, impurity control, shelf life, and storage conditions must be addressed for translation (Théry, 2018).

Thus, a more feasible route is to use exosomes as the model of ideal delivery and a conceptual framework, while developing scalable biomimetic carriers (e.g., exosome-like nanovesicles, membrane-wrapped nanoparticles) to replicate their key advantages at the engineering level (Jang et al., 2013). At the same time, stricter quality control measures (e.g., particle size distribution, protein/lipid markers, miRNA loading levels, functional potency assays) should be introduced to meet requirements in clinical development (Théry, 2018). Therefore, although exosomes provide a biologically compelling model for follicular miRNA delivery, future development may require engineered exosome-like nanovesicles or membrane-coated nanoparticles with standardized particle characterization, miRNA-loading quantification, potency assays, and batch-release criteria.

### 5.4 Physical enhancement delivery: microneedles, iontophoresis, ultrasound, electroporation

For deep hair follicle targets (bulge HFSC and DP), physical enhancement is often required for adequate penetration to reach the target, and remains a clinically feasible approach (Prausnitz and Langer, 2008).

Microneedles can directly penetrate the stratum corneum, delivering nucleic acids or carriers to the subepidermal/dermal superficial layers, significantly improving delivery. Dissolvable microneedles enable local deposition and sustained release, coated microneedles are convenient for polypharmaceutical applications, and hollow microneedles offer more precise dosage control. Microneedle advantages include repeatability, localization, and precise control of dose. However, limitations include complex preparation, potential pain, local inflammation risks, and adherence challenges for large-scale scalp application.

Iontophoresis uses an electric field to drive charged molecules across the skin. This technique improves local permeability and can be combined with gel-based patches. However, its efficiency for delivering large molecules and to deeper tissue targets is limited (Kalia et al., 2004).

Ultrasound and electroporation temporarily alter barrier structures or cell membrane permeability, enhancing uptake. However, they require careful attention to safety, repeated dosing tolerance, and equipment standardization (Prausnitz and Langer, 2008).

In hair regeneration, physical enhancement strategies serve more as a “delivery switch.” They do not replace carriers but can significantly expand effective delivery, allowing low-dose nucleic acids to achieve therapeutic effects while simultaneously reducing inflammation and toxicity risks.

### 5.5 Delivery and efficacy evaluation criteria

To translate delivery systems from research to clinical application, a robust, reproducible system of evaluation must be established. The advantages and disadvantages of different delivery systems vary (Table 3). It is recommended to use a four-dimensional evidence framework: 1) Spatial Reach (Biodistribution/Localization): A potential therapy must demonstrate that the delivery system enters the hair follicle unit and localizes to target compartments (bulge, DP, or adjacent regions), rather than merely remaining at the skin surface (Blume-Peytavi and Vogt, 2011). 2) Mechanistic Engagement: A potential therapy must prove that the mimic completes RISC loading and induces downregulation of key target genes (or that anti-miRs achieve functional miRNA silencing), which is core evidence of an effective delivery (Hu B. et al., 2020). 3) Phenotypic Readout: A potential therapy must demonstrate hair follicle cycle progression (e.g., telogen to anagen ratio changes), improvements in hair density/diameter, hair shaft quality, etc. (Dhurat and Saraogi, 2009). 4) Safety and Tolerability: A potential therapy must demonstrate low local inflammation scores, barrier function indicators, changes in immune cell profiles, and repeated dosing tolerance. For the AA scenario, immune endpoint monitoring is particularly critical (Yano et al., 2001; Gilhar et al., 2012).

**TABLE 3:** Comparative horizontal overview of hair follicle targeting delivery platforms: Penetration depth, cell occupancy efficiency, immune risk, and CMC feasibility.

Delivery platform/Technology	Penetration depth (depth to hair follicle unit)	Cell occupancy efficiency (distribution within target compartment)	Immune risk and safety	CMC feasibility (manufacturing and scalability)	Representative literature
Liposome	Medium: Reaches subepidermal layers, surface layers of the follicle	Low to Medium: Sometimes penetrates the follicle base, but often limited to the epidermal layer	Low: Natural liposomes have low immunogenicity	High: Conventional methods can scale to large-scale production	Blume-Peytavi and Vogt (2011)
Exosome	High depth: Can penetrate hair follicles through intercellular gaps and adhesion forces	High: Exosomes effectively enter hair follicles, with strong cell specificity	Low: Exosomes have natural low immunogenicity, reducing immune response	High: Small-scale production is relatively simple, but large-scale production is limited by extraction efficiency	Hu et al. (2020b)
Nanoparticles	High depth: Can penetrate deep into the hair follicle and enter through the follicle opening	Medium to High: Good dispersion and strong aggregation towards the hair follicle	Moderate: Without modification, may cause local inflammation or cytotoxicity	Low: Some types may not meet large-scale production requirements	Dhurat and Saraogi (2009)
Polymeric Microparticles	Medium: Penetrates the epidermis to reach the base of the hair follicle	Medium: Particle size can be adjusted for specific follicular regions	Low: Surface modification can reduce immune response, low toxicity	High: Good scalability and batch production capabilities	Yano et al. (2001)
Gene Gun	High: Creates small pores in the epidermis, reaching the hair follicle region	High: Can achieve deep targeting through mechanical means	Higher: Penetration may induce local inflammation	Low: Large-scale application limited by cost and equipment	Gilhar et al. (2012)
Microneedle Arrays	High: Directly penetrates the stratum corneum to reach the bottom of the hair follicle	High: Effective release into the follicle area	Low: Microneedles are small, with minimal impact on local immune response	High: Suitable for mass production, with low equipment costs	Hu et al. (2020b)
Ultrasound	Adjustable depth: Enhances penetration through physical means, reaching deep hair follicles	Medium: Enhanced delivery with ultrasound, but dependent on wave frequency and dose	Moderate: Risk of immune response not fully eliminated	Low: Suitable for small-scale experiments, with challenges in large-scale production	Yano et al. (2001)

## 6 Translational challenges

Although miRNAs and their mimics have shown potential in promoting hair regeneration, improving the microenvironment, and regulating immune thresholds in cell and animal models, transitioning them from experimental models to clinical application requires overcoming a series of translational hurdles that are commonly shared among all nucleic acid-based drugs (Khvorova and Watts, 2017). The uniqueness of hair regeneration lies in the fact that the target organ is located on the skin surface, making it suitable and simplistic for local delivery. However, this relative ease in target organ access is offset by a strong physical barrier, an extensive immune surveillance system, and the need for long-cycle phenotypic outputs (Oh et al., 2016; Olsen et al., 2004). Therefore, translational evaluation must extend from a sequence-based pathway to a system framework that can be reviewed for the following characteristics: 1) Spatial localization: Does the delivery system reach key target compartments (e.g., HFSC/DP/immune cells)? 2) Temporal coverage: Does the timing align with the hair follicle cycle window (Oh et al., 2016)? 3) Safety boundary: Can repeated dosing be sustained long-term (Dolte et al., 2000)? 4) CMC and batch consistency: Is scalability achievable (Khvorova and Watts, 2017)? 5) Clinical endpoints: Are endpoints standardized and auditable (Lindow and Kauppinen, 2012)?

The translational challenge for miRNA mimics is not a single technical issue but rather a system engineering problem comprised of multi-target network effects, skin immune and barrier environments, cycle-driven temporal output, CMC consistency, and verifiable clinical endpoints (Table 4). For a marketable pathway, the most critical strategy should emphasize modular mechanism-driven selection of candidate miRNAs and combination strategies; mechanistic readouts that are interpretable and span preclinical and clinical stages; standardized endpoints and long-term safety boundaries that define sustainable efficacy. With these strategic components, any new miRNA-based therapy should be able to seamlessly display clear evidence of efficacy that is reviewable, products that are reproducible, and risks that are manageable.

**TABLE 4:** miRNA hair regeneration therapy translational risk registry: key risks, minimum validation set, mitigation strategies, and reversibility.

Key risks	Minimum validation set	Mitigation strategies	Reversibility	Representative literature
Multi-target Network Effect Risk	miRNA candidate screening: Screening and validating the action networks of candidate miRNAs through *in vitro*/animal models	Select modular mechanism-driven miRNA combinations to reduce non-specific targeting and increase predictability of therapeutic effects	Medium: Therapy strategies can be gradually optimized by adjusting dose and combination, fine-tuning treatment duration	Hu et al. (2020b), Deleavey and Damha (2012)
Skin Immune Response and Barrier Damage	Local immune response assessment: Skin irritation, inflammation scores, immune cell profile changes, local tolerance testing	Moderate chemical modifications such as 2′-O modification, moderate phosphorothioate modification to reduce immune activation; use low-immunogenicity materials	High: Immune responses can be adjusted by modifying the delivery system composition and dosage	Robbins et al. (2007), Capaldi et al. (2017)
Cycle-driven Temporal Output Issues	Hair growth cycle monitoring: Hair density, hair diameter, follicle morphology, and other objective traits; regular imaging and counting	Include long-term observation periods in clinical trial design to ensure sustained efficacy and avoid short-term reaction false positives	Medium: Efficacy evaluation can be gradually collected during clinical follow-up stages and treatment strategies adjusted	Dhurat and Saraogi (2009)
CMC Consistency and Manufacturing Challenges	Production batch stability assessment: Consistency of miRNA modification types, delivery vehicle quality and stability	Optimize production processes and standardize protocols to ensure batch stability and reproducibility, minimizing production variability	Medium: Manufacturing process improvements and process validation can ensure a degree of reversibility	Crooke et al. (2020)
Clinical Endpoint Verifiability	Clinical endpoint data collection: Objective morphological data (hair density, hair diameter), patient-reported outcomes (PRO), quality of life assessments	Use standardized clinical endpoint measurement methods to ensure comparability and verifiability of data; multi-center data collection through cross-institutional collaboration	High: Ensuring transparency and reproducibility of data collection and clinical endpoint assessment	Olsen et al. (2004)

## 7 Mechanism combination application strategy and future directions for disease scenarios

Hair regeneration disorders exhibit significant clinical heterogeneity. Although all involve hair loss, AGA is more related to androgen-driven regression of the DP microenvironment and follicular miniaturization (Chen et al., 2025), AA is driven by immune privilege disruption and inflammatory gate failure (Bertolini et al., 2020), while chemotherapy-induced hair loss is typically driven by rapid proliferative cell damage and apoptosis (Hasl et al., 2021). Therefore, the application of miRNAs and their mimics should not be limited to the myopic point of view that any specific miRNA promotes growth. Instead, miRNA-based therapeutic development should focus on constructing mechanism combinations at the module level. This means that therapies within the same disease context should simultaneously address (1) lowering inhibition thresholds (anti-regression/anti-inflammation/immune silencing), and (2) enhancing regenerative drive (Wnt/Shh/DP induction), and 3) implementing delivery strategies to achieve adequate spatial targeting and temporal coverage. More translatable combinations often require a third element—sustainability. This requires that therapies achieve long-term net effects with low doses, repeatable and controllable interventions, without excessive stimulation.

## 8 Clinical landscape and future perspectives

At present, the clinical translation of mechanism-defined miRNA therapeutics for hair regeneration remains at an early stage. Publicly available clinical-trial registries show that several exosome- or small extracellular vesicle-based approaches are being investigated for androgenetic alopecia, including studies evaluating exosome treatment, human umbilical cord mesenchymal stem cell-derived small extracellular vesicles, and exosome-based interventions compared with platelet-rich plasma. However, these trials should not be directly interpreted as clinical trials of defined miRNA mimics or miRNA inhibitors, because naturally derived vesicle products contain complex mixtures of miRNAs, proteins, lipids, and other bioactive molecules. Their therapeutic activity cannot be attributed to a specific miRNA unless the active cargo, dose, potency, and target engagement are experimentally validated.

Therefore, future miRNA-based hair-regeneration therapies may develop along two complementary routes. The first route involves biologically derived or engineered vesicle products, in which pro-regenerative miRNAs are enriched or selectively loaded into exosomes or exosome-mimetic vesicles. The second route involves chemically defined miRNA mimics or inhibitors delivered by LNPs, polymeric nanoparticles, microneedles, or other local delivery systems. Experience from non-dermatological miRNA therapeutics, such as liposomal miR-34a mimic development in oncology, suggests that miRNA replacement therapy is biologically feasible but also highlights the importance of immune safety, dose control, and rigorous pharmacodynamic validation (Hong et al., 2020).

For hair regeneration, future clinical studies should clearly distinguish between vesicle-based regenerative products and mechanism-defined miRNA drugs. Essential translational requirements include selection of pathway-relevant miRNA candidates, confirmation of target engagement in hair follicle stem cells or dermal papilla cells, alignment of dosing schedules with the hair cycle, standardized assessment of hair density and hair-shaft diameter, and long-term monitoring of local inflammation, fibrosis, and off-target tissue effects.

## 9 Conclusion

Hair regeneration is essentially a branched, network-based regenerative process driven by the activation of HFSCs, the restoration of DP inductive capacity, and the rebalancing of the immune-stromal microenvironment. Its success depends on whether the anagen initiation threshold can be effectively lowered and maintained in a stable state (Diener et al., 2022). As key post-transcriptional network regulators, miRNAs can integrate multiple targets, including Wnt/β-catenin, Shh, TGF-β/BMP, AR-related regression signals, and inflammatory pathways (e.g., IFN-γ/JAK-STAT and NF-κB). Thus, they offer a programmable and combinable entry point for restarting the regeneration network (Chen et al., 2025).

In terms of therapeutic strategies, miRNA mimics enhance pro-regenerative signaling, while anti-miRs/ASOs help diminish regressive or pro-inflammatory signals. Together, they form a complementary pro-regeneration and anti-regression/immune remodeling mechanistic framework, which aligns better with the pathophysiological features of hair loss seen with AGA and AA (Chen et al., 2025). For clinical heterogeneity, a more translatable rationale could include de-noising inflammation and lowering thresholds, ignition to promote initiation, and low-frequency maintenance for sustainability, rather than strong single-point stimulation. For AGA, the pathological core is more related to androgen-driven follicular regression and miniaturization, while for AA, it is the interruption of the hair cycle due to immune privilege breakdown and failure of inflammatory gating (Kim et al., 2025).

At the same time, the key translational barriers focus on off-target effects, network side effects, local immunogenicity and barrier tolerance, dose-timing alignment with the hair follicle cycle, and the consistency and scalability of the delivery system and CMC quality control (Makkar, 2025). Future developmental efforts should be guided by modular mechanisms to screen candidate miRNAs and design combination therapies, focusing on overcoming follicular-targeted delivery and engineered time-based dosing. A standardized evaluation system should be established throughout preclinical and clinical stages, covering spatial targeting, mechanistic engagement, phenotypic consistency, and safety boundaries. By translating mechanistic advantages into interpretable, reproducible, and reviewable evidence, miRNAs and their mimics will remain a provocative area for hair regeneration treatment, shifting from empirical stimulation to precise network regulation.
